# Association of 9-Valent HPV Vaccination with Risk of Juvenile Idiopathic Arthritis: A Sex-Stratified Real-World Study

**DOI:** 10.3390/vaccines14090749

**Published:** 2026-08-28

**Authors:** Yi-Sin Liou, Lin-Shien Fu, Hsin-Hua Chen, Wen-Yu Wu, Yung-Chieh Huang, Chiann-Yi Hsu, Ming-Chin Tsai

**Affiliations:** 1 Department of Pediatrics, Taichung Veterans General Hospital, 1650, Section 4, Taiwan Boulevard, Taichung 40705, Taiwan; 2 Department of Post-Baccalaureate Medicine, National Chung Hsing University, Taichung 407219, Taiwan; 3 Department of Medicine, Taichung Veterans General Hospital, Taichung 407219, Taiwan; 4 Center for Quality Management, Taichung Veterans General Hospital, Taichung 407219, Taiwan; 5 Rong Hsing Research Center for Translational Medicine, National Chung Hsing University, Taichung 407219, Taiwan; 6 Big Data Center, National Chung Hsing University, Taichung 407219, Taiwan; 7 Doctoral Program in Translational Medicine, National Chung Hsing University, Taichung 40227, Taiwan; 8 Department of Medical Research, Taichung Veterans General Hospital, Taichung 407219, Taiwan

**Keywords:** juvenile idiopathic arthritis, HPV9 vaccination, sex differences, immune mechanisms

## Abstract

Objective: To evaluate the association between 9-valent human papillomavirus (HPV9) vaccination and the risk of new-onset juvenile idiopathic arthritis (JIA), with particular attention to sex-specific differences, including the relative lack of evidence in males and the potential impact of the COVID-19 pandemic. Patients and Methods: Using the TriNetX U.S. Collaborative Network (2016–2023), children aged 9–13 years who received HPV9 vaccination were compared with unvaccinated controls in prepandemic and pandemic periods. Incident JIA was assessed using matched cohort analyses and time-to-event methods. Results: Similar sex-specific patterns were observed across both periods. HPV9 vaccination was associated with a lower risk of JIA among girls (HR = 0.45 95% CI = 0.22–0.95 in prepandemic and HR = 0.36 95% CI = 0.17–0.74 during pandemic period), while no increased risk was observed among boys (HR = 1.57 95% CI = 0.67–4.05 in prepandemic and HR = 1.14 95% CI = 0.56–2.34 during pandemic period). Among unvaccinated individuals, girls consistently exhibited a higher incidence of JIA than boys. The COVID-19 pandemic did not alter these associations. Conclusions: HPV9 vaccination was not associated with an increased risk of JIA in either sex. A consistent sex-specific pattern was observed across both study periods, with a lower risk of JIA among vaccinated girls but not boys. This finding suggests a potential sex difference in the association between HPV9 vaccination and JIA and warrants further investigation. However, residual confounding, including healthy-vaccinee bias and differences in healthcare utilization, cannot be fully excluded.

## 1. Introduction

Human papillomavirus (HPV) infection is among the most common viral infections worldwide and is a well-established cause of cervical, anogenital, and oropharyngeal cancers [1,2]. In addition to malignancy, HPV is responsible for benign conditions such as genital warts, contributing to a substantial global disease burden [3]. Prophylactic HPV vaccination has markedly reduced the incidence of HPV-related diseases [4,5]. The introduction of the 9-valent HPV vaccine (HPV9), approved by the U.S. Food and Drug Administration in 2014, expanded protection to additional oncogenic HPV types [6]. Widespread implementation of HPV9 vaccination programs since 2016 has created an opportunity to examine vaccine safety as well as broader immune-related outcomes in large real-world populations.

Juvenile idiopathic arthritis (JIA) is the most common chronic inflammatory rheumatic disease in childhood and represents a heterogeneous group of autoimmune disorders characterized by persistent synovial inflammation [7,8]. In the United States, the prevalence of JIA has been estimated at approximately 13.2 to 30.2 per 100,000 children [9]. Epidemiologically, JIA demonstrates a consistent female predominance across many populations, suggesting that sex-related biological factors contribute to disease susceptibility [10,11]. More broadly, many autoimmune diseases exhibit a strong sex bias, highlighting the importance of interactions between immune regulation, hormonal influences, and environmental exposures in shaping autoimmune risk. However, whether vaccine-related autoimmune outcomes differ by sex—particularly in males—remains insufficiently explored.

Viral infections have long been implicated as potential triggers of autoimmune diseases through mechanisms such as molecular mimicry, bystander activation, and immune dysregulation. From this perspective, vaccination against common viral pathogens provides a unique population-level context in which to examine how controlled antigen exposure may interact with host factors—including sex—to influence autoimmune susceptibility.

Concerns regarding autoimmune diseases following vaccination have prompted multiple epidemiologic investigations, particularly focusing on JIA. The HPV9 vaccine contains amorphous aluminum hydroxyphosphate sulfate (AAHS) as an adjuvant to enhance the immune response to HPV virus-like particles. Current evidence has not established that adjuvant increases the risk of autoimmune disease [12]. Overall, existing studies have not demonstrated an increased risk of JIA after HPV vaccination. In a prior population-based analysis using the TriNetX network, we observed no elevation in JIA incidence among HPV4-vaccinated adolescent females; notably, vaccination was associated with a lower risk compared with unvaccinated peers [13]. However, evidence in male adolescents remains limited, largely reflecting the later inclusion of boys in HPV vaccination programs.

With the expanded use of HPV9 vaccination in both males and females since 2016, this study analyzed a large real-world cohort to examine sex-specific associations between HPV9 vaccination and subsequent development of JIA. Specifically, the primary analysis evaluated the association between HPV9 vaccination and subsequent JIA risk separately in males and females. Secondary analyses compared JIA risk between males and females within the vaccinated and unvaccinated populations. Temporal analyses were performed to assess whether these associations were consistent across two independent post-approval periods (2016–2019 and 2020–2023), spanning the prepandemic and COVID-19 pandemic eras.

## 2. Methods

### 2.1. Data Source

This retrospective cohort study was conducted using the TriNetX Research Network (TriNetX, LLC, Cambridge, MA, USA). The database includes information on demographics, diagnoses, procedures, medications, and vaccinations and is compliant with the U.S. Health Insurance Portability and Accountability Act (HIPAA) [14]. TriNetX has been widely used for large-scale epidemiologic and pharmacoepidemiologic studies using real-world clinical data. Because only de-identified, aggregate-level data were analyzed, informed consent was not required. Institutional review board approval was obtained at Taichung Veterans General Hospital (IRB No. CE23454A), consistent with institutional requirements for studies using secondary de-identified data.

### 2.2. Study Population and Study Design

**Figure 1:**
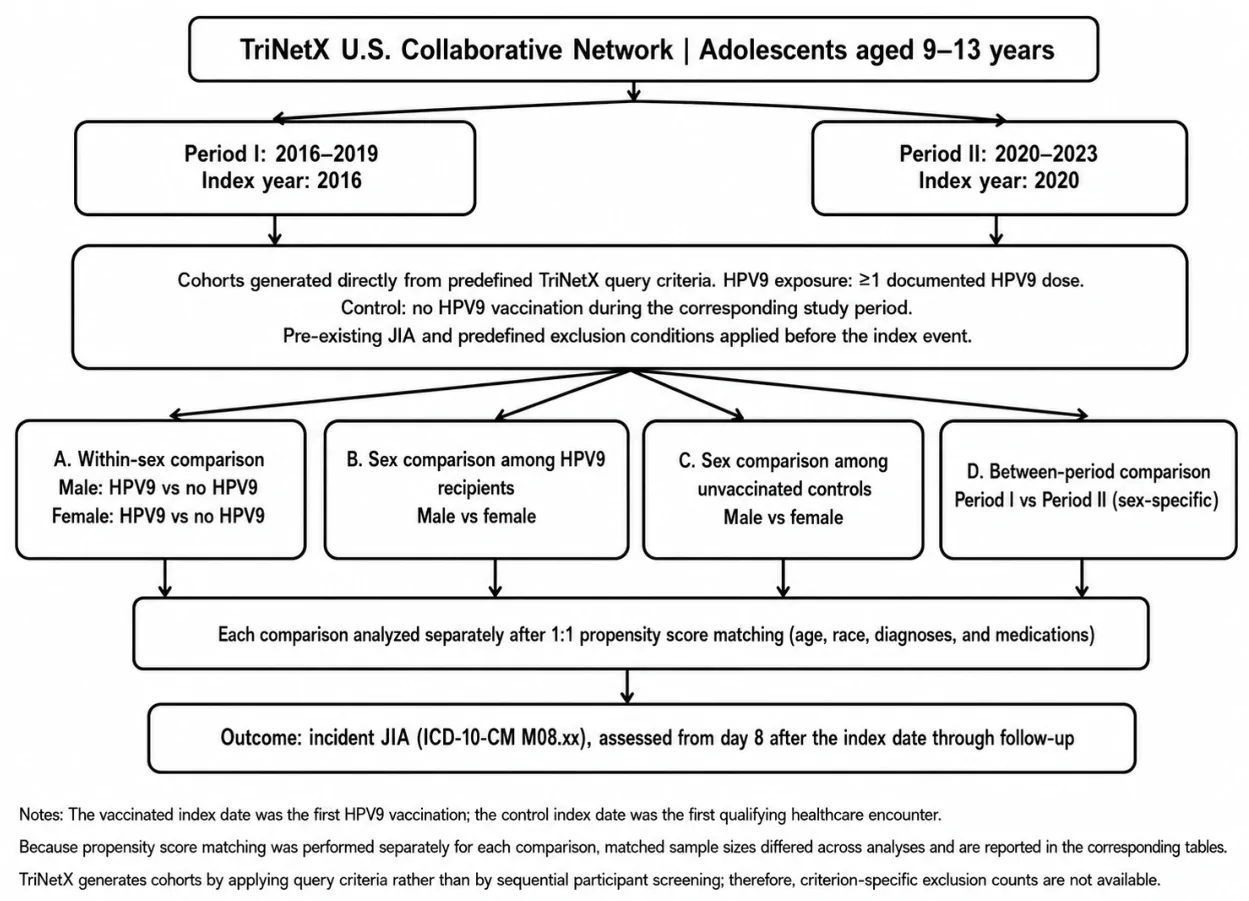
Study Design and Analysis Framework.

Two independent study periods were defined to reflect HPV9 vaccine uptake before and during the COVID-19 pandemic: 2016–2019 and 2020–2023. Participants aged 9 to 13 years at the index date were eligible for inclusion. The index date was defined as the date of first documented 9-valent human papillomavirus (HPV9) vaccination for vaccinated individuals and a matched healthcare visit date for unvaccinated controls. Participants in the control group were required to have no documented HPV9 vaccination throughout the corresponding study period (2016–2019 or 2020–2023). The study design and analytical framework are summarized in Figure 1.

The age range was selected to ensure that all participants remained younger than 16 years throughout follow-up, consistent with the diagnostic definition of juvenile idiopathic arthritis (JIA). Individuals with a documented diagnosis of juvenile idiopathic arthritis prior to the index date were excluded. To further ensure incident case ascertainment, patients with evidence of rheumatoid factor positivity or prior methotrexate use before the index date were also excluded.

### 2.3. Data Management

Exposure was defined as receipt of at least one dose of the 9-valent human papillomavirus vaccine, identified using standardized vaccination codes within the TriNetX platform. The primary outcome was incident juvenile idiopathic arthritis, defined by the presence of ICD-10-CM diagnostic codes M08.xx. A 7-day lag period was applied, with JIA outcomes assessed beginning on day 8 after the index date. Follow-up extended for up to three years after the index date, with censoring at the end of follow-up or loss to follow-up. Demographic and clinical variables used for cohort construction, exclusion criteria, and propensity score matching included age, sex, race, relevant musculoskeletal conditions, rheumatoid-factor positivity, prior HPV vaccination, and relevant medication use. Definitions and corresponding TriNetX codes for the exposure, outcome, and explanatory variables are provided in Supplementary Table S1.

### 2.4. Statistical Analysis

Descriptive analyses were performed to summarize baseline demographic and clinical characteristics. Continuous variables were summarized using means and standard deviations, and categorical variables were summarized using counts and percentages. Standardized mean differences (SMDs) were used to assess covariate balance before and after propensity score matching.

Baseline covariates were balanced between HPV9-vaccinated participants and unvaccinated controls using 1:1 propensity score matching (greedy nearest-neighbor) with a caliper of 0.1 on the logit of the propensity score. Propensity score matching was performed independently for each predefined comparison, including the primary HPV9-versus-unvaccinated comparisons within each sex, the secondary male-versus-female comparisons within vaccination strata, and the between-period comparisons. Because matching was performed separately for each analysis, matched sample sizes could differ across comparisons. Covariate balance after matching was assessed using SMDs, with values <0.1 considered indicative of adequate balance.

Incident JIA was assessed using the built-in TriNetX Compare Outcomes analytics. Participants with JIA documented before the index date were excluded using the TriNetX “exclude patients with the outcome prior to the time window” setting. A 7-day lag period was applied, with outcome assessment beginning on day 8 after the index date. Follow-up extended for up to 2.5 years after the index date, and cumulative probabilities of JIA were evaluated at prespecified follow-up time points.

Cumulative probabilities of JIA were estimated using the TriNetX survival analysis. Hazard ratios (HRs) and 95% confidence intervals (CIs) were generated using the Cox proportional hazards analysis implemented within the TriNetX platform. The proportional hazards assumption was assessed using the proportionality test provided by the TriNetX Survival module. Patients were censored after the last recorded fact in their electronic health record, according to the predefined TriNetX survival-analysis procedure.

Missing data were not imputed by the investigators. Analyses were based on data available within the TriNetX network, and no patient-level data were accessed or independently analyzed by the investigators. All analyses were conducted within the TriNetX analytics platform (TriNetX, LLC, Cambridge, MA, USA).

## 3. Results

### 3.1. Propensity Score Matching and Baseline Characteristics (Table 1)

Table 1 summarizes the study cohort construction and the sample sizes before and after propensity score matching (PSM) for each prespecified analysis. The primary analyses compared HPV9-vaccinated participants with unvaccinated controls separately in males and females within each study period. The secondary analyses compared males with females within the vaccinated and unvaccinated populations. Temporal analyses compared corresponding cohorts between Period I and Period II. Each comparison was matched independently; therefore, matched cohort sizes differed across analyses. Detailed baseline characteristics and covariate balance before and after PSM are provided in Supplementary Table S2, with standardized mean differences (SMDs) used to assess covariate balance.

**Table 1:** Study Cohorts and Propensity Score-Matched Analytic Comparisons.

Analysis	Study Period	Comparison	Before PSM, *n*	After 1:1 PSM, *n*
**Primary analysis:** **HPV9–JIA association**	Period I (2016–2019)	Male: HPV9 vs. no HPV9	25,184 vs. 624,641	25,184 vs. 25,184
	Period I (2016–2019)	Female: HPV9 vs. no HPV9	22,661 vs. 598,022	22,650 vs. 22,650
**Secondary analysis:** **sex comparison**	Period I (2016–2019)	HPV9: Male vs. Female	25,184 vs. 22,661	21,920 vs. 21,920
	Period I (2016–2019)	No HPV9: Male vs. Female	624,641 vs. 598,022	598,022 vs. 598,022
**Primary analysis:** **HPV9–JIA association**	Period II (2020–2023)	Male: HPV9 vs. no HPV9	35,724 vs. 723,108	35,724 vs. 35,724
	Period II (2020–2023)	Female: HPV9 vs. no HPV9	34,746 vs. 642,375	34,746 vs. 34,746
**Secondary analysis:** **sex comparison**	Period II (2020–2023)	HPV9: Male vs. Female	35,724 vs. 34,746	33,610 vs. 33,610
	Period II (2020–2023)	No HPV9: Male vs. Female	723,108 vs. 642,375	642,375 vs. 642,375
**Temporal analysis:** **between-period comparison**	Period I vs. II	Male, no HPV9	624,641 vs. 723,108	624,641 vs. 624,641
	Period I vs. II	Female, no HPV9	598,022 vs. 642,375	598,022 vs. 598,022

Note. PSM was performed independently for each comparison; therefore, matched cohort sizes may differ across analyses. Detailed baseline characteristics and covariate balance are provided in Supplementary Table S2. Primary analyses compared HPV9-vaccinated participants with unvaccinated controls within each sex and study period; sex and temporal comparisons were secondary analyses.

Detailed baseline characteristics and covariate balance before and after PSM are provided in Supplementary Table S2. All included covariates achieved adequate post-matching balance (SMD < 0.1). The corresponding JIA outcome analyses are presented in Table 2A,B and in the temporal comparison, described below.

### 3.2. Period I: 2016–2019 Cohort (Table 2A)

After matching, all included covariates achieved adequate balance, with standardized mean differences < 0.1 across the comparisons. The matched cohorts were subsequently used for the corresponding outcome analyses presented in Supplementary Table S2A–C. Period I: 2016–2019 cohort (Table 2A).

The 2016–2019 cohort included participants who received HPV9 vaccination or had a clinical visit in 2016 and who were followed for up to 30 months.

### 3.3. Primary Analysis: HPV9 Vaccination and JIA Risk

Among males, HPV9 vaccination was not associated with a significantly increased risk of JIA compared with unvaccinated controls (HR, 1.57; 95% CI, 0.67–4.05). Among females, HPV9 vaccination was associated with a significantly lower risk of JIA compared with unvaccinated controls (HR, 0.45; 95% CI, 0.22–0.95).

### 3.4. Secondary Analysis: Sex Comparisons

Among HPV9-vaccinated participants, JIA risk did not differ significantly between males and females (HR, 0.78; 95% CI, 0.19–3.13). Among unvaccinated participants, females had a higher incidence of JIA than males (HR for males vs. females, 0.74; 95% CI, 0.65–0.83), consistent with the known female predominance in JIA susceptibility.

### 3.5. Period II: 2020–2023 Cohort (Table 2B)

The 2020–2023 cohort included adolescents with HPV9 vaccination or healthcare encounters in 2020 and were followed for up to 2.5 years under pandemic conditions. After PSM, baseline characteristics remained well balanced between vaccinated and unvaccinated groups in both sexes (Supplementary Table S2D–F).

### 3.6. Primary Analysis: HPV9 Vaccination and JIA Risk

Among males, HPV9 vaccination was not associated with a significantly increased risk of JIA compared with unvaccinated controls (HR, 1.14; 95% CI, 0.56–2.34). Among females, HPV9 vaccination was associated with a significantly lower risk of JIA compared with unvaccinated controls (HR, 0.36; 95% CI, 0.17–0.74).

### 3.7. Secondary Analysis: Sex Comparisons

Among HPV9-vaccinated participants, JIA risk did not differ significantly between males and females (HR, 0.92; 95% CI, 0.40–2.08). Among unvaccinated participants, females had a higher incidence of JIA than males (HR for males vs. females, 0.55; 95% CI, 0.47–0.64).

### 3.8. Temporal Analysis: Comparison Between Study Periods

For the between-period comparison of HPV9-vaccinated participants, PSM yielded 19,607 matched males and 19,745 matched females for each study period. However, because of the small number of incident JIA events in the HPV9-vaccinated cohorts, TriNetX did not provide sufficient data for a direct between-period comparison or reliable estimation of HRs. Therefore, between-period HRs for HPV9-vaccinated males and females are not reported.

For participants without HPV9 vaccination, PSM yielded 624,641 males and 598,022 females in each study period. The incidence of JIA was lower during 2020–2023 than during 2016–2019 in both males (250 vs. 354 JIA events; HR, 0.71; 95% CI, 0.60–0.83) and females (480 vs. 609 JIA events; HR, 0.79; 95% CI, 0.70–0.89).

Taken together, these findings indicate that HPV9 vaccination was not associated with an increased risk of JIA in either sex, while an inverse association with JIA was consistently observed among vaccinated females across the two cohorts. The persistence of higher baseline JIA risk among unvaccinated females across both periods was consistent with the known female predominance in JIA susceptibility.

No statistically significant violation of the proportional hazards assumption was detected in the Cox analyses for Period I, Period II, or the between-period comparisons (all proportionality test *p*-values > 0.05).

## 4. Discussion

This large real-world cohort study using the TriNetX Research Network demonstrates a sex-specific association between HPV9 vaccination and juvenile idiopathic arthritis (JIA) risk. HPV9 vaccination was not associated with an increased risk of JIA in either sex, while vaccinated females showed a significantly lower 3-year cumulative incidence compared with unvaccinated controls. These findings were highly consistent across two independent post-approval periods (2016–2019 and 2020–2023). These findings provide reassuring evidence regarding the risk of JIA following HPV9 vaccination.

HPV9 received U.S. FDA approval in late 20 [14], and large-scale implementation began in 2016 [6]. Because JIA is defined as onset before 16 years of age [15], we restricted follow-up to three years after vaccination to ensure comparability across age groups and to capture early autoimmune events potentially occurring after vaccination.

As shown in Table 2, the overall incidence of JIA among unvaccinated controls was lower in 2020–2023 than in 2016–2019 for both sexes. This temporal decline likely reflects, at least in part, pandemic-related changes in pediatric healthcare utilization, including reduced outpatient visits and deferred non-urgent consultations [16,17]. In addition, public health measures such as physical distancing, school closures, and mask use substantially reduced circulation of common respiratory viruses, including influenza and RSV [18,19], which have been proposed as potential triggers of autoimmune activation. Pandemic-related disruptions have also been associated with reduced circulation of several viral pathogens and altered healthcare utilization patterns, which may partly explain the lower baseline JIA incidence observed during 2020–2023. While not directly generalizable, such observations underscore how large-scale public health disruptions may influence patterns of viral exposure and detection, providing contextual support for the lower baseline JIA incidence observed among unvaccinated individuals during 2020–2023.

Against this backdrop of reduced baseline risk, the sex-specific association between HPV9 vaccination and subsequent JIA remained consistent across the two study periods. In both the 2016–2019 and 2020–2023 cohorts, HPV9 vaccination was not associated with an increased risk of JIA in either sex and was consistently linked to a lower risk among females.

The reproducibility of these sex-specific associations across two calendar periods with differing baseline JIA incidence suggests that the observed pattern is unlikely to be solely explained by period-dependent factors, such as changes in healthcare access or short-term fluctuations in infection exposure. Although the later cohort coincided with the COVID-19 pandemic era, we did not observe any evidence that the study period modified the direction or magnitude of the HPV9–JIA association. Formal testing for period-by-sex interaction was limited by the rarity of incident JIA events after matching. Nonetheless, the parallel findings across two independently constructed cohorts support the interpretation that more stable host-related factors—particularly sex-based immune regulation—play a central role in shaping autoimmune susceptibility following HPV antigen exposure.

Sex-based immune differences are a fundamental determinant of autoimmune susceptibility. Approximately 80% of autoimmune diseases occur in females, reflecting the combined influences of sex hormones, genetic factors, and epigenetic regulation [10,11,20]. Estrogen enhances B-cell activation and type I interferon production, while androgens suppress inflammatory cytokines and promote regulatory T-cell differentiation [20,21,22,23,24]. Experimental data show that estrogen upregulates TLR7 and TLR9 expression—both responsive to VLP components—and enhances IL-10 and regulatory T-cell activity, which may favor immune tolerance rather than auto-reactivity [25,26]. Comprehensive reviews have also emphasized that females show lower thresholds for B-cell activation and impaired Treg homeostasis compared with males [27]. These mechanisms explain the higher JIA prevalence in unvaccinated females observed in both 2016 and 2020 cohorts.

HPV vaccination, while primarily prophylactic, involves virus-like particle (VLP) antigens that can modulate innate immune pathways. Evidence from HPV4 trials indicates that females mount higher antibody titers and longer-lasting seropositivity than males across all genotypes [28]. In this study, vaccinated females—but not males—showed reduced JIA risk, suggesting a possible sex-dependent immunomodulatory effect. Experimental data show that estrogen upregulates TLR7 and TLR9 expression—both responsive to VLP components—and enhances IL-10 and regulatory T-cell activity, which may favor immune tolerance rather than auto-reactivity [23,27,29,30]. In contrast, androgen-driven immune suppression may dampen such recalibration in males, resulting in the neutral effect observed.

To date, no population-based study has provided comparable real-world evidence on sex-stratified autoimmune outcomes following HPV vaccination. Our results provide additional real-world data on sex-stratified autoimmune outcomes following HPV vaccination and highlight the need for further studies specifically designed to investigate these sex-related differences. Further studies integrating cytokine, transcriptomic, and hormonal data are warranted to elucidate the mechanistic basis of these differences. Importantly, given the relative lack of prior data in males, our findings provide additional real-world evidence to help address this gap and support a more balanced understanding of vaccine-related autoimmune risk across sexes.

Several limitations merit consideration. Despite robust matching and large sample sizes, residual confounding cannot be excluded because potentially relevant factors, including genetic background, family history of autoimmune disease, environmental exposures, previous infections, vaccination adherence, and healthcare-seeking behavior, were not fully available in TriNetX; moreover, the direction and magnitude of their influence cannot be determined. No formal correction for multiple comparisons was applied, and the possibility of type I error, particularly in subgroup analyses, should therefore be considered when interpreting these findings as exploratory. In addition, index events differed between groups, with vaccination defining the index date for vaccinated participants and a healthcare encounter for controls, potentially resulting in differences in healthcare utilization or diagnostic opportunity. The predominantly U.S.-based population may also limit generalizability. Finally, mechanistic data, including cytokine dynamics and autoantibody profiles, were unavailable. The consistency of the observed patterns across the two study periods nevertheless supports further investigation in studies with more detailed clinical and behavioral information.

## 5. Conclusions

HPV9 vaccination was not associated with an increased risk of JIA in either sex. An inverse association with JIA was consistently observed among vaccinated females across both study periods; however, this finding should be considered exploratory because residual confounding, including healthy-vaccinee bias and differences in healthcare utilization, cannot be excluded. These findings provide reassuring evidence regarding JIA risk following HPV9 vaccination and highlight the importance of further investigation into potential sex-related differences in vaccine-associated immune responses.

## Figures and Tables

**Table 2:** (**A**) Cumulative probabilities (%) and hazard ratio of JIA since Index Day during 2016–2019. (**B**) Cumulative probabilities (%) and hazard ratio of JIA since Index Day during 2020–2023.

(A)								
		Number with Outcome (at 36 Months)	6M (%)	12M (%)	24M (%)	30M (%)	HR (95%CI)	Forest Plot (HR, 95% CI)
Male							1.57 (0.67−4.05)	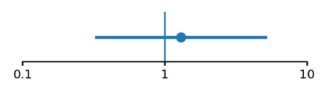
HPV9	25,184	11	0	0.008	0.036	0.045		
No HPV9	25,184	7	0	0.005	0.030	0.035		
Female							0.45 (0.22−0.95)	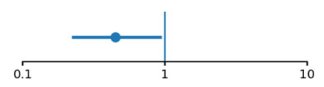
HPV9	22,650	10	0	0	0.034	0.048		
No HPV9	22,650	22	0.0526	0.053	0.091	0.097		
Male							0.78 (0.19−3.13)	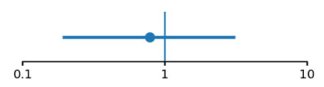
HPV9	21,920	8	0.005	0.01	0.03	0.04		
Female								
HPV9	21,920	10	0	0.005	0.035	0.05		
Male							0.74 (0.65−0.83)	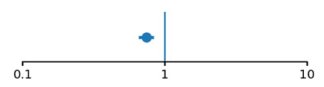
No HPV9	598,022	299	0.02	0.035	0.04	0.05		
Female								
No HPV9	598,022	508	0.014	0.032	0.074	0.085		
(**B**)								
		**Number with Outcome (at 36 Months)**	**6M (%)**	**12M (%)**	**24M (%)**	**30M (%)**	**HR (95%CI)**	**Forest plot (HR, 95% CI)**
Male							1.14 (0.56–2.34)	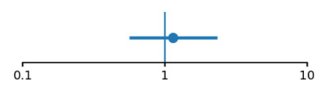
HPV9	35,724	16	0	0.006	0.036	0.045		
No HPV9	35,724	14	0	0.006	0.031	0.039		
Female							0.36 (0.17–0.74)	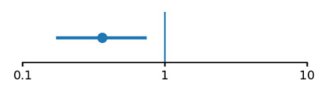
HPV9	34,746	17	0	0	0.035	0.049		
No HPV9	34,746	41	0.035	0.055	0.092	0.12		
Male							0.92 (0.40–2.08)	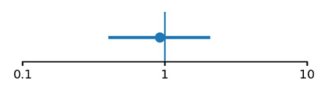
HPV9	33,610	11	0.002	0.006	0.03	0.036		
Female								
HPV9	33,610	12	0	0.007	0.035	0.039		
Male							0.55 (0.47–0.64)	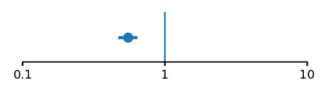
No HPV9	642,375	259	0.016	0.026	0.04	0.04		
Female								
No HPV9	642,375	473	0.023	0.032	0.059	0.074		

Dots represent hazard ratios (HRs), and horizontal lines represent 95% confidence intervals (CIs). The vertical line at HR = 1.

## Data Availability

The data used in this study were obtained from the TriNetX Research Network. Due to data use agreements and patient privacy regulations, the datasets are not publicly available. Access to the data is available through TriNetX under appropriate institutional agreements.

## Supplementary Materials

The following supporting information can be downloaded at https://www.mdpi.com/article/10.3390/vaccines14090749/s1. Table S1: Codes related in this study; Table S2: baseline characteristics.
