# Supplementary material for: Association of 9-Valent HPV Vaccination with Risk of Juvenile Idiopathic Arthritis: A Sex-Stratified Real-World Study
Source: Vaccines (Basel). 2026 Aug 28;14(9):749. doi: 10.3390/vaccines14090749 (PMC13611020; doi:10.3390/vaccines14090749)
Supplement: Supplementary file 1 [file vaccines-14-00749-s001.zip › vaccines-4482181-supplementary.pdf]

Supplementary data

Table S1 Codes related in this study:

Diagnosis:

- UMLS:ICD10CM:M08 :Juvenile Idiopathic Arthritis (JIA)
- UMLS:ICD10CM:M00-M99: Diseases of the musculoskeletal system and connective tissue
- UMLS:ICD10CM:R76.0 : Raised antibody titer
- UMLS:ICD10CM:U07.1: COVID-19

Medication:

- NLM:VA:MS102: NONSALICYLATE NSAIDs,ANTIRHEUMATIC
- NLM:RXNORM:6851: Methotrexate
- NLM:VA:MS190: ANTIRHEUMATICS,OTHER

Procedures:

- UMLS:CPT:90649: Human Papillomavirus vaccine, types 6, 11, 16, 18, quadrivalent (4vHPV), 3 dose schedule, for intramuscular use
- UMLS:CPT:90650: Human Papillomavirus vaccine, types 16, 18, bivalent (2vHPV), 3 dose schedule, for intramuscular use
- UMLS:CPT:90651: Human Papillomavirus vaccine types 6, 11, 16, 18, 31, 33, 45, 52, 58, nonavalent (9vHPV), 2 or 3 dose schedule, for intramuscular use

Laboratory :

- TNX:9070: Rheumatoid factor [Units/volume] in Serum or Plasma (at least 20.00 [IU]/mL)
- TNX:9088: SARS coronavirus 2 and related RNA [Presence]
- TNX:9089: SARS coronavirus 2 IgG IgM Ab [Presence] in Serum or Plasma
- UMLS:LNC:41458-1: SARS coronavirus RNA [Presence] in Specimen by NAA with probe detection
- UMLS:LNC:41460-7: SARS coronavirus IgG Ab [Presence] in Serum by Immunoassay
- UMLS:LNC:94558-4: SARS-CoV-2 (COVID-19) Ag [Presence] in Respiratory system specimen by Rapid immunoassay
- UMLS:LNC:94562-6: SARS-CoV-2 (COVID-19) IgA Ab [Presence] in Serum or Plasma by Immunoassay
- UMLS:LNC:94762-2: SARS-CoV-2 (COVID-19) Ab [Presence] in Serum or Plasma by Immunoassay

- UMLS:LNC:94769-7: SARS-CoV-2 (COVID-19) Ab [Units/volume] in Serum or Plasma by Immunoassay
- UMLS:LNC:95209-3: SARS-CoV+SARS-CoV-2 (COVID-19) Ag [Presence] in Respiratory system specimen by Rapid immunoassay
- UMLS:LNC:96603-6: SARS-CoV-2 (COVID-19) S protein RBD neutralizing antibody [Presence] in Serum or Plasma by sVNT
- UMLS:LNC:96119-3: SARS-CoV-2 (COVID-19) Ag [Presence] in Upper respiratory specimen by Immunoassay
- UMLS:LNC:97097-0: SARS-CoV-2 (COVID-19) Ag [Presence] in Upper respiratory specimen by Rapid immunoassay

Table S2A Baseline characteristics (male 2016, Before COVID-19 pandemic)

	Before propensity score matching						After propensity score matching					
	HPV9		Control		P-Value	SMD	HPV9		Control		P-Value	SMD
	Patients	SD;%	Patients	SD;%			Patients	SD;%	Patients	SD;%		
No. of individuals	22,408		695,306				22,408		22,408			
Age at Index (years)	11.6	±0.9	10.8	±1.4	<0.001	0.665	11.6	±0.9	11.6	±0.9	1.000	<0.001
Race												
White	11,992	53.5%	413,046	59.4	<0.001	0.119	11,992	53.5%	11,992	53.5%	1.000	<0.001
Black or African American	4,836	21.6%	113,698	16.4	<0.001	0.134	4,836	21.6%	4,836	21.6%	1.000	<0.001
Asian	879	3.9%	24,092	3.5%	0.001	0.024	879	3.9%	879	3.9%	1.000	<0.001
Diagnosis												
M00-M99	4,688	20.9%	83,371	12.0%	<0.001	0.243	4,688	20.9%	4,688	20.9%	1.000	<0.001
L40.5	0	0%	25	0.0%	0.369	0.008	0	0%	0	0%	--	--
Medication (MS102)	4,759	21.2%	103,001	14.8%	<0.001	0.168	4,759	21.2%	4,759	21.2%	1.000	<0.001

M00-M99: diseases of the musculoskeletal system and connective tissue; L40.5: arthropathic psoriasis; MS102: NONSALICYLATE NSAIs,ANTIRHEUMATIC; SDM standardized mean difference.

Table S2B Baseline characteristics (Female 2016, Before COVID-19 pandemic)

	Before propensity score matching						After propensity score matching					
	HPV9		Control		P-Value	SMD	HPV9		Control		P-Value	SMD
	Patients	SD;%	Patients	SD;%			Patients	SD;%	Patients	SD;%		
No. of individuals	22,661		680,797				22,661		22,661			
Age at Index (years)	11.6	±0.9	10.9	±1.5	<0.001	0.585	11.6	±0.9	11.6	±0.9	1.000	<0.001
Race												
White	12,193	53.8%	405,390	59.5%	<0.001	0.116	12,193	53.8%	12,193	53.8%	1.000	<0.001
Black or African American	4,773	21.1%	110,366	16.2%	<0.001	0.125	4,773	21.1%	4,773	21.1%	1.000	<0.001
Asian	878	3.9%	23,644	3.5%	0.001	0.021	878	3.9%	878	3.9%	1.000	<0.001
Diagnosis												
M00-M99	5,251	23.2%	97,169	14.3%	<0.001	0.230	5,251	23.2%	5,251	23.2%	1.000	<0.001
L40.5	0	0%	35	0.0%	0.280	0.010	0	0%	0	0%	1.000	<0.001
Medication (MS102)	4,860	21.4%	101,491	14.9%	<0.001	0.170	4,860	21.4%	4,860	21.4%	1.000	<0.001

M00-M99: diseases of the musculoskeletal system and connective tissue; L40.5: arthropathic psoriasis; MS102: NONSALICYLATE NSAIs,ANTIRHEUMATIC; SDM standardized mean difference.

Table S2C Baseline characteristics (Male VS Female, Before COVID-19 pandemic)

	**Before propensity score matching**						**After propensity score matching**					
	Male-Control		Female-Control		P-Value	SMD	Male-Control		Female-Control		P-Value	SMD
	Patients	SD;%	Patients	SD;%			Patients	SD;%	Patients	SD;%		
No. of individuals	695,306		657,797				653,468		653,468			
Age at Index (years)	10.8	±1.4	10.9	±1.5	<0.001	0.029	10.9	±1.5	10.9	±1.5	0.662	0.001
Race												
White	413,046	59.4%	392,876	59.7%	<0.001	0.007	389,243	59.6%	389,783	59.6%	0.336	0.002
Black or African American	113,698	16.4%	107,545	16.3%	0.963	<0.001	107,470	16.4%	106,994	16.4%	0.261	0.002
Asian	24,092	3.5%	23,298	3.5%	0.015	0.004	23,083	3.5%	23,058	3.5%	0.906	<0.001
Diagnosis												
M00-M99	83,371	12.0%	86,761	13.2%	<0.001	0.036	82,595	12.6%	82,434	12.6%	0.672	0.001
L40.5	25	0%	34	0.0%	0.166	0.002	19	0%	33	0%	0.052	0.003
Medication (MS102)	103,001	14.8%	95,934	14.6%	<0.001	0.006	95,299	14.6%	95,187	14.6%	0.498	<0.001

M00-M99: diseases of the musculoskeletal system and connective tissue; L40.5: arthropathic psoriasis; MS102: NONSALICYLATE NSAIs,ANTIRHEUMATIC; SDM standardized mean difference.

Table S2D Baseline characteristics (Male 2020, During COVID-19 pandemic)

	Before propensity score matching						After propensity score matching					
	HPV9		Control		P-Value	SMD	HPV9		Control		P-Value	SMD
	Patients	SD;%	Patients	SD;%			Patients	SD;%	Patients	SD;%		
No. of individuals	35,120		708,646				35,120		35,120			
Age at Index (years)	11.4	±0.9	10.8	±1.5	<0.001	0.501	11.4	±0.9	11.4	±0.9	1.000	<0.001
Race												
White	18,920	53.9%	413,617	58.4%	<0.001	0.091	18,920	53.9%	18,920	53.9%	1.000	<0.001
Black or African American	6,747	19.2%	109,819	15.5%	<0.001	0.098	6,747	19.2%	6,747	19.2%	1.000	<0.001
Asian	1,702	4.8%	26,053	3.7%	<0.001	0.058	1,702	4.8%	1,702	4.8%	1.000	<0.001
Diagnosis												
M00-M99	8,894	25.3%	130,848	18.5%	<0.001	0.166	8,894	25.3%	8,894	25.3%	1.000	<0.001
L40.5	0	0%	22	0.0%	0.296	0.008	0	0%	0	0%	1.000	<0.001
Medication (MS102)	10,806	30.8%	173,190	24.4%	<0.001	0.142	10,806	30.8%	10,806	30.8%	1.000	<0.001

M00-M99: diseases of the musculoskeletal system and connective tissue; L40.5: arthropathic psoriasis; MS102: NONSALICYLATE NSAIs,ANTIRHEUMATIC; SDM standardized mean difference.

Table S2E Baseline characteristics (Female 2020, During COVID-19 pandemic)

	Before propensity score matching						After propensity score matching					
	HPV9		Control		P-Value	SMD	HPV9		Control		P-Value	SMD
	Patients	SD;%	Patients	SD;%			Patients	SD;%	Patients	SD;%		
No. of individuals	34,839		701,202				34,839		34,839			
Age at Index (years)	11.4	±0.9	10.9	±1.5	<0.001	0.427	11.4	±0.9	11.4	±0.9	1.000	<0.001
Race												
White	18,799	54.0%	405,848	57.9%	<0.001	0.079	18,799	54.0%	18,799	54.0%	1.000	<0.001
Black or African American	6,811	19.5%	112,707	16.1%	<0.001	0.091	6,811	19.5%	6,811	19.5%	1.000	<0.001
Asian	1,701	4.9%	24,878	3.5%	<0.001	0.066	1,701	4.9%	1,701	4.9%	1.000	<0.001
Diagnosis												
M00-M99	9,388	26.9%	138,471	19.7%	<0.001	0.171	9,388	26.9%	9,388	26.9%	1.000	<0.001
L40.5	0	0%	38	0.0%	0.169	0.010	0	0%	0	0%	1.000	<0.001
Medication (MS102)	10,464	30.0%	165,583	23.6%	<0.001	0.145	10,464	30.0%	10,464	30.0%	1.000	<0.001

M00-M99: diseases of the musculoskeletal system and connective tissue; L40.5: arthropathic psoriasis; MS102: NONSALICYLATE NSAIs,ANTIRHEUMATIC; SDM standardized mean difference.

Table S2F Baseline characteristics (Male VS Female, During COVID-19 pandemic)

	Before propensity score matching						After propensity score matching					
	Male-Control		Female-Control		P-Value	SMD	Male-Control		Female-Control		P-Value	SMD
	Patients	SD;%	Patients	SD;%			Patients	SD;%	Patients	SD;%		
No. of individuals	646,116		614,929				609,383		609,383			
Age at Index (years)	10.8	±1.4	10.9	±1.5	<0.001	0.035	10.9	±1.5	10.9	±1.5	0.441	0.001
Race												
White	373,896	57.9%	354,798	57.7%	0.052	0.003	351,017	57.6%	351,389	57.6%	0.495	0.001
Black or African American	105,445	16.3%	101,358	16.5%	0.013	0.004	100,569	16.5%	100,405	16.5%	0.689	0.001
Asian	24,180	3.7%	22,557	3.7%	0.028	0.004	22,584	3.7%	22,470	3.7%	0.584	0.001
Diagnosis												
M00-M99	117,543	18.2%	120,440	19.6%	<0.001	0.036	115,606	19.0%	115,189	18.9%	0.335	0.002
L40.5	22	0%	35	0.0%	0.056	0.003	15	0%	15	0%	1.000	<0.001
Medication (MS102)	143,681	23.4%	155,212	24.0%	<0.001	0.015	142,946	23.5%	142,629	23.4%	0.498	0.001

M00-M99: diseases of the musculoskeletal system and connective tissue; L40.5: arthropathic psoriasis; MS102: NONSALICYLATE NSAIs,ANTIRHEUMATIC; SDM standardized mean difference.
